# Mitochondrial DNA variation and phylogeography of Old World camels

**DOI:** 10.5713/ajas.20.0319

**Published:** 2020-08-24

**Authors:** Liang Ming, Dalai Siren, Li Yi, Le Hai, Jing He, Rimutu Ji

**Affiliations:** 1 Key Laboratory of Dairy Biotechnology and Bioengineering, Ministry of Education, College of Food Science and Engineering, Inner Mongolia Agricultural University, Hohhot 010018, China; 2 Camel Research Institute of Inner Mongolia, Alashan 737300, China

**Keywords:** Bactrian Camel, Dromedary, Haplotypes, Genetic Diversity, Population Expansion

## Abstract

**Objective:**

Old World camels are a valuable genetic resource for many countries around the world due to their adaptation to the desert environment. At present, Old World camels have encountered the challenge of unprecedented loss of genetic resources. Through our research, we would reveal the population structure and genetic variation in Old World camel populations, which provides a theoretical basis for understanding the germplasm resources and origin and evolution of different Old World camel populations.

**Methods:**

In the present study, we assessed mtDNA control region sequences of 182 individuals from Old World camels to unravel genetic diversity, phylogeography, and demographic dynamics.

**Results:**

Thirty-two haplotypes confirmed by 54 polymorphic sites were identified in the 156 sequences, which included 129 domestic and 27 wild Bactrian camels. Meanwhile, 14 haplotypes were defined by 47 polymorphic sites from 26 sequences in the dromedaries. The wild Bactrian camel population showed the lowest haplotype and nucleotide diversity, while the dromedaries investigated had the highest. The phylogenetic analysis suggests that there are several shared haplotypes in different Bactrian camel populations, and that there has been genetic introgression between domestic Bactrian camels and dromedaries. In addition, positive values of Tajima’s D and Fu’s *F*s test demonstrated a decrease in population size and/or balancing selection in the wild Bactrian camel population. In contrast, the negative values of Tajima’s D and Fu’s *F*s test in East Asian Bactrian camel populations explained the demographic expansion and/or positive selection.

**Conclusion:**

In summary, we report novel information regarding the genetic diversity, population structure and demographic dynamics of Old World camels. The findings obtained from the present study reveal that abundant genetic diversity occurs in domestic Bactrian camel populations and dromedaries, while there are low levels of haplotype and nucleotide diversity in the wild Bactrian camel population.

## INTRODUCTION

The Old World camels are represented by the Tylopoda suborder which comprises domesticated Bactrian camel (*Camelus Bactrianus*) and dromedary (*Camelus dromedarius*), and the only representative wild species remaining, wild Bactrian camel (*Camelus ferus*), following the extinction of the wild dromedary [1]. Traditionally, the Old World camels have provided a wide range of useful products to Gobi desert rural communities, including meat, milk, and wool, since their domestication around 3,000 to 6,000 years ago. Currently, the high nutritional and medical value of their milk has increased the number of breeders, who have extended the exploitation of these species to other regions outside the Gobi Desert, especially Bactrian camels. The natural habitat of the domestic Bactrian camel are the cold desert areas of Northeast and Central Asia, in contrast to the dromedaries, which are adapted to the semi-arid and hot desert regions of North Africa and East Africa, the Arabian Peninsula and Southwest Asia [2]. The wild Bactrian camel is restricted to few remaining refuge areas, with their range extending to only three locations in China (Taklamakan desert, Gashun Gobi Desert and Arjin Mountains in the Lop Nur Lake region) and one in Mongolia (Great Gobi Strictly Protected Area ‘A’) [3]. Nowadays, the wild Bactrian camel is listed as Critically Endangered [4] and its population is estimated to number from a few hundred to 2,000 individuals [5,6]. The Old World camels show several biological and physiological traits that may be connected with adaptation to such extreme heat and harsh and dry environments, including resistance to hunger and thirst, fluctuating body temperatures [7], tolerance of a high dietary intake of salt, and an immune system producing unique immunoglobulin [8]. Recent genomic studies have revealed positive selection [9] and immune gene loci, which may be related to desert environmental adaptation in this species [10].

Mitochondrial DNA (mtDNA), as a molecular marker, is widely used in the identification of wild ancestors and maternal lines that have contributed to a breed or population [11–14]. Over recent years, nuclear and mtDNA markers have been used intensively to unravel the genetic diversity and phylogeographic structure in the Old World camels [15–17]. Within mtDNA, the control region (CR) has been revealed to evolve five times faster than the coding region [18]. Therefore, the CR is considered suitable for assessing population genetic structure, variability and phylogeny.

With the modernization of society and increasing desertification, the genomic resources from the two domesticated Old World camel species, dromedary (*Camelus dromedarius*) and Bactrian camel (*Camelus bactrianus*), and one wild species, wild Bactrian camel (*Camelus Ferus*), have been seriously affected; this is especially the case for the wild Bactrian camel species, which is on the verge of extinction [4]. Unfortunately, little is known about the genetic diversity and phylogeography of Old World camels. Therefore, we selected mtDNA CR sequences of 182 individuals from domestic and wild Bactrian camels, and dromedaries, to investigate the genetic diversity, phylogeography and demographic dynamics of the different camel populations.

## MATERIALS AND METHODS

### Data sources

The present study was conducted on 182 sequences which were downloaded from GenBank. These included wild Bactrian camel from Mongolia (n = 27); domestic Bactrian camel from China (n = 57), Mongolia (n = 43), Russia (n = 10), Kazakhstan (n = 6), and Iran (n = 13); and dromedary (n = 26) from Africa (Supplementary Table S1). In accordance with references [15,17,19], we restricted the 809 bp mtDNA fragment nt 15120–15928 from each sequence, and multiple sequence alignments were performed using Mega version 6 [20] against the wild Bactrian camel reference sequence (Genbank accession number NC_009628) for Bactrian camel populations, and the dromedary reference sequence (Genbank accession number NC_009849) for dromedaries.

### Genetic diversity estimation

DnaSP v5 was used to determine the level of genetic diversity, including polymorphic sites (S), number of haplotypes (H), haplotype diversity (Hd), nucleotide diversity (π), the average number of nucleotide differences (K) and the standard deviations (SD). These parameters were identified for each population and across all populations [21].

### Phylogenetic analysis

In order to assess the possible genetic relationship between individuals and populations, a phylogenetic tree was constructed with the neighbor-joining (NJ) algorithm implemented in MEGA6 [20]. The confidence level for each branch was evaluated with 1,000 bootstrap replications. To further infer the genetic relationship between the haplotypes from different camel populations, a median-joining (MJ) network was built using the NETWORK 5.0.0 with equal weight mutations and character states [22].

### Neutrality test and demographic dynamics

We calculated the demographic profiles for each geographic region from mismatch distribution patterns [23]. At the same time, the Harpending’s raggedness index “r” [24] statistic was used to evaluate the significance of the deviations of the observed sum of squares differences (SSD) from the simulated model of expansion based on 1,000 coalescent simulations. Furthermore, Fu’s *F*s [25] and Tajima’s D [26] statistics were also considered using the infinite sites model in Arlequin v3.5 [27]. Tajima’s D is negative, indicating that there is an excess of low-frequency polymorphisms relative to expectations, and the population size is expanding (both bottlenecks or selective sweep) or purification selection is occurring [28]. Positive Tajima’s D signifies low levels of high frequency polymorphisms, explaining a decrease in population size and/or balancing selection. The same holds for Fu’s *F*s test, which is more powerful than Tajima’s D [29].

## RESULTS

### mtDNA sequence haplotype variation and genetic diversity

Thirty-two haplotypes confirmed by 54 polymorphic sites were identified in the 156 sequences, which included 129 domestic Bactrian camels and 27 wild Bactrian camels. There were 30 haplotypes determined in the domestic Bactrian camel population and two in the wild Bactrian camel population. Meanwhile, 14 haplotypes were defined by 47 polymorphic sites from 26 sequences in the dromedaries (Table 1, Supplementary Figure S1, S2). In the Bactrian camel populations, six haplotypes were shared by at least two populations (Supplementary Table S2), and the most frequent haplotype was H_4 (29%; 45 sequences out of 156), followed by haplotype H_3 (13%; 21 sequences out of 156). Several haplotypes included H_9, which was present only in samples from Russia (n = 5), and H_18, which was present only in samples from China (n = 2). Two specific haplotypes (W_1 and W_2) were determined in the extant wild Bactrian camel populations. In the dromedaries, the commonest haplotypes were D_1 and D_9, representing 27% and 15% of all sequences, respectively (Supplementary Table S2; Figure S2).

At the country level, excepting the wild Bactrian camel populations, all other populations showed a high level of maternal genetic diversity, including dromedaries. In the domestic Bactrian camel populations, the highest level of Hd (0.876±0.033) was observed in Mongolia, while the lowest (0.600±0.0215) was found in Kazakhstan; the lowest level of nucleotide diversity (0.00188) was observed in China, while the highest (0.066) was observed in Iran’s domestic Bactrian camel population.

Furthermore, we divided domestic Bactrian camel populations into two groups to investigate genetic diversity: East Asia (China and Mongolia’s domestic Bactrian camel populations) and Central Asia (Iran, Kazakhstan and Russia’s domestic Bactrian camel populations). The number of haplotypes from east Asia (H = 21) was higher than central Asia (H = 13); however, the genetic diversity for populations from Central Asia (Hd = 0.896±0.033 and π = 0.01043) was slightly higher than East Asia (Hd = 0.801±0.030 and π = 0.00238). Interestingly, the wild Bactrian camel population showed the lowest Hd (0.462±0.065) and nucleotide diversity (0.00115), while dromedaries revealed the highest Hd (0.908±0.041) in this study (Table 1).

### Population phylogenetic analysis

**Figure 1:**
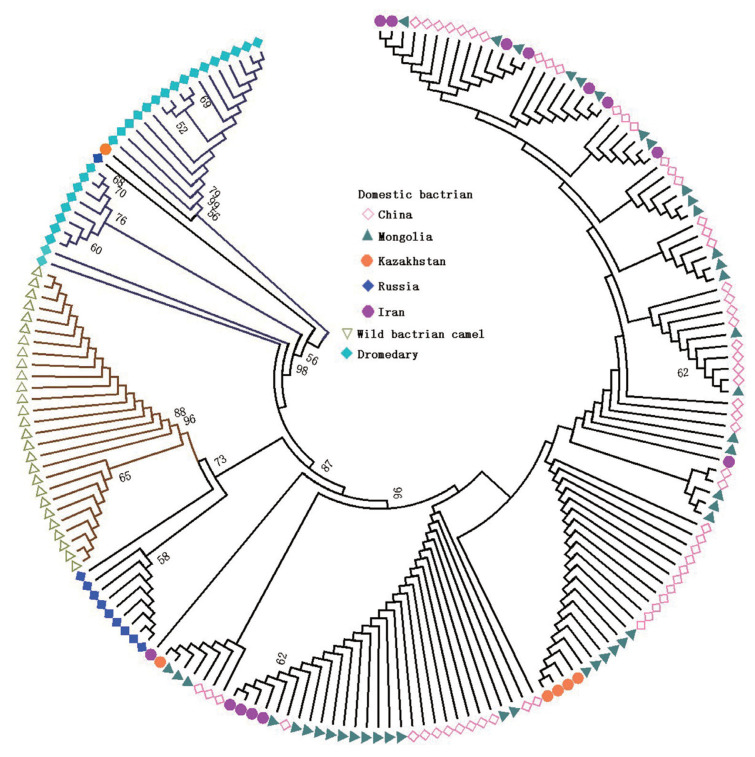
Neighbor-joining tree constructed using the control region (809 bp) of the mtDNA of domestic and wild Bactrian camel populations, and dromedaries. Different colors and shape marks indicate different camel populations, including domestic bactrian camel (from China, Mongolia, Kazakhstan, Russia, and Iran), wild bactrian camel and dromedary. Wild bactrian camels and dromedaries were clustered separately, and there is almost no geographical difference between the different Bactrian camel populations.

In order to assess genetic relationships between Bactrian camels and dromedaries, the NJ tree was constructed for the 809 bp CR (Figure 1) and haplotype sequences (Supplementary Figure S3). The NJ tree revealed three well-resolved clusters, namely, domestic Bactrian camels, wild Bactrian camels and dromedaries (Figure 1). Interestingly, two individuals from Kazakhstan (H_2, MH109985) and Russia (H_8, MH109974) were clustered with the dromedaries, which indicated that introgression of mtDNA CR existed between Bactrian camels and dromedaries.

**Figure 2:**
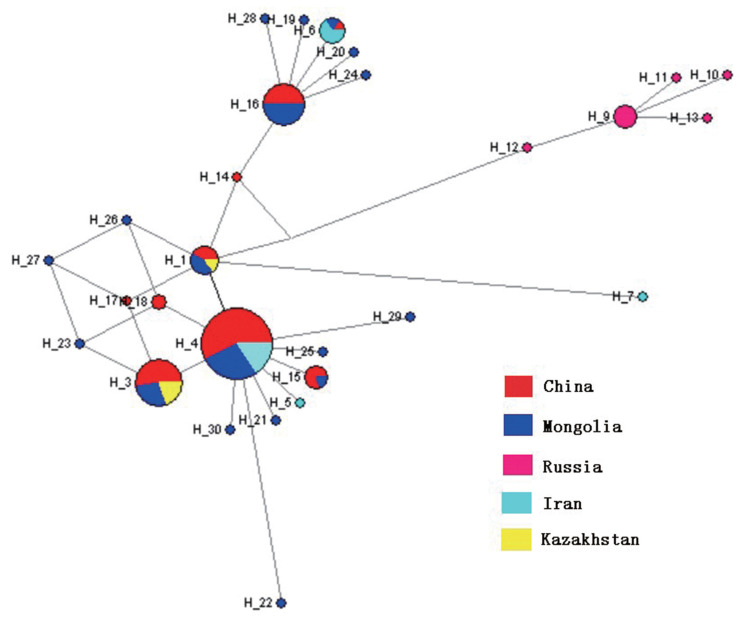
Median-joining network for domestic Bactrian camel population haplotypes from China, Mongolia, Russia, Iran and Kazakhstan. The area of the circles are proportional to the haplotype frequency. Haplotypes of each populations from different geographic regions, such as China, Mongolia, Iran, and Kazakhstan did not cluster together according to their geographic regions.

Furthermore, the phylogenetic relationships among domestic Bactrian camel population haplotypes, with H_2 and H_8 excluded, are shown in a MJ network (Figure 2). Haplotypes of each population from different geographic regions (China, Mongolia, Iran, and Kazakhstan) did not cluster together according to their geographic regions. Several common haplotypes, such as H_1, H_3, H_4 and H_20, were shared by individuals from different geographic regions. These results indicated that there was no correspondence between the geographic regions of origin and relationships among populations. All haplotypes networks are shown in Supplementary Figure S4. This result clearly showed that all camel haplotypes were divided into three distinct lineages, similar to the findings from the NJ tree. Meanwhile, for haplotypes H_2 (MH109985) and H_8 (MH109974) from the Bactrian camels, rather than clustering with the Bactrian camel groups, they were closer to the Iranian dromedary. This indicated that there was introgression between Bactrian camels from Russia and Kazakhstan and Iranian dromedary.

### Population history and demographic dynamics

We predicted mismatch distribution patterns for domestic Bactrian camel populations from different geographic regions, the wild Bactrian camel population and the dromedary population. To clarify the demographic dynamics of Old World camels, the mismatch distribution graphs revealed uni-, bi- and multi-modal patterns (Supplementary Figure S5), which suggest that there has been a demographic expansion, bottleneck or purifying selection in different camel populations. The above results were compatible with the calculation of population demographic parameters from Table 2. Negative non-significant Tajima’s D values were observed for almost all domestic Bactrian camel populations, with the exception of the Russian Bactrian camel, for which Tajima’s D was negative but significant (p<0.01). The wild Bactrian camel population and dromedaries showed positive and non-significant Tajima’s D values. The test of Tajima’s D is used to distinguish between a DNA sequence evolving randomly (neutrally) and one evolving under a non-random process, including directional selection or balancing selection, demographic expansion or contraction, genetic hitchhiking, or introgression [26]. The Central Asian domestic Bactrian camel populations, the wild Bactrian camel population and dromedaries exhibited positive and non-significant Fu’s *F*s values. These results suggest a complex demographic history for the different camel populations. While no significant differences were found for the Harpending raggedness index (r) and SSD in Old World camel populations, the results demonstrated that our total dataset showed relatively good fit to the population expansion model [30].

## DISCUSSION

In this study, we assessed the mtDNA genetic diversity of 182 Old World camels including domestic Bactrian camels from China, Mongolia, Russia, Iran and Kazakhstan, wild Bactrian camel from Mongolia; and dromedaries. The aim of our research would reveal Old World camel populations history and demographic dynamics using the most polymorphic CR in mtDNA. This highly polymorphic CR has been used in previous research to assess the genetic diversity and phylogeny of camels, especially in relation to the maternal origin of Old World Camels [15,17,19], including the Bactrian camels.

We observed higher haplotype and nucleotide diversity in the dromedary CRs compared to the domestic and wild Bactrian camel populations (Table 1). This corresponds to previous estimates on whole mitochondrial genome sequencing, and the haplotype and nucleotide diversity of domestic (0.952±0.096) and wild Bactrian camels (0.822±0.097) are lower than dromedary (1.0±0.045) [12]. The wider geographical distribution of the domestic Bactrian camel, which come from China, Mongolia, Kazakhstan, Russia and Iran, and the dromedaries, which come from Iran, Saudi Arabia, United Arab Emirates, Kenya, Sudan, Pakistan and Morocco, could in principle explain these differences. In contrast, the wild Bactrian camel population originated from only one region (the Strictly Protected Area “A” in the Mongolian Gobi Desert), which may restrict its genetic diversity. In addition, researchers believe that the small size of the wild Bactrian camel population, with a census indicating as few as 2,000 individuals remaining [6], is also an important reason for their low genetic diversity [12,15,31]. Similar to previous results [19,15,31], only two haplotypes were discovered (W_1 and W_2) in the wild Bactrian camel population, which again showed its low genetic diversity. A possible caveat of our study is that CR (809 bp) sequence variation alone might not be sufficient to recover population genetic divergence in the wild Bactrian camel population.

In the domestic Bactrian camel populations, there was no distinguishing geographic structuring in the NJ tree and median-joining network among populations from China, Mongolia, Russia, Kazakhstan, and Iran. This result indicated the populations’ single maternal lineage and that strong gene introgression existed in different domestic Bactrian camel populations, especially individuals MH109985 (H_2) and MH109974 (H_8) from Kazakhstan and Russia, which were clustered with the dromedaries; this finding has also been confirmed in whole genome sequencing research [32]. The Silk Road could, in principle, account for this introgression circumstance.

During the process of domestication, population growth and dispersion of animals across a wider geographic range can be determined from molecular signals of sudden expansion [8]. In our study, we obtained positive values of Tajima’s D and Fu’s *F*s test in the wild Bactrian camel population, which demonstrates a decrease in population size and/or balancing selection. At the same time, positive values of Fu’s *F*s test were found in the Central Asian (i.e., from Russia, Kazakhstan, and Iran) domestic Bactrian camels, which may also be related to a decreasing population size, especially considering that an Iranian population census indicated there were just several hundred individuals remaining. These results are in agreement with reduced genetic diversity and small effective population size in wild and domestic Iranian Bactrian camels. Meanwhile, negative values of Tajima’s D and Fu’s *F*s test in East Asian populations (i.e., from China and Mongolia) of domestic Bactrian camels can be explained by demographic expansion and/or positive selection.

## CONCLUSION

In summary, we report novel information regarding genetic diversity, population structure and demographic dynamics of Old World camels. The findings obtained from the present study reveal that abundant genetic diversity occurs in domestic Bactrian camel populations and dromedaries, while low levels of haplotype and nucleotide diversity were found in the wild Bactrian camel population.

## Figures and Tables

**Table 1:** Genetic diversity of Bactrian camel and dromedary populations from the analysis of the control region

Category	Geographical region	N	S	H	Hd±SD	π	k
Domestic bactrian camel	China	57	7	9	0.738±0.047	0.00188	1.518
	Mongolia	43	17	18	0.876±0.033	0.00298	2.394
	Kazakhstan	6	24	3	0.600±0.0215	0.01043	8.400
	Russia	10	24	6	0.778±0.137	0.00638	5.133
	Iran	13	12	5	0.808±0.066	0.066	0.004
	Central Asia1)	29	39	13	0.896±0.033	0.01043	8.387
	East Asia2)	100	17	21	0.801±0.030	0.00238	1.909
	All domestic3)	129	49	30	0.833±0.025	0.00459	3.677
Wild Bactrian camel	Mongolia	27	2	2	0.462±0.065	0.00115	0.923
Dromedary	Africa	26	47	14	0.908±0.041	0.02323	18.726

N, sample size; S, number of polymorphic sites; H, number of haplotypes; Hd, haplotype diversity; SD, standard deviation; π, nucleotide diversity; K, average number of nucleotide differences.

1) Central Asia, domestic Bactrian camel from Kazakhstan, Russia and Iran.

2) East Asia, domestic Bactrian camel from China and Mongolia.

3) All domestic, domestic Bactrian camel from China, Mongolia, Kazakhstan, Russia and Iran.

**Table 2:** Population demographic parameters estimated from the analysis of the control region of the mtDNA in camel populations

Population	N	S	SSD (p-value)	Hapending’s r	Tajima’s D (p-value)	Fu’s *F*s (p-value)
China	57	7	0.012 (0.100)	0.067 (0.130)	−0.050 (0.584)	−0.195 (0.178)
Mongolia	43	16	0.007 (0.47)	0.031 (0.600)	−1.159 (0.086)	−9.281 (0.001)
Kazakhstan1)	5	2	0.172 (0.000)	0.680 (0.900)	−0.973 (0.094)	1.040 (0.610)
Russia	10	24	0.054 (0.130)	0.187 (0.470)	−1.876 (0.005)	0.450 (0.606)
Iran	13	12	0.042 (0.45)	0.084 (0.540)	−0.401 (0.303)	1.366 (0.832)
Central Asia	29	39	0.030 (0.270)	0.045 (0.260)	−0.5750 (0.259)	0.266 (0.591)
East Asia	100	17	0.011 (0.380)	0.041 (0.480)	−1.1826 (0.087)	−12.294 (0.000)
Wild	27	2	0.173 (0.070)	0.716 (0.050)	1.648 (0.899)	3.024 (0.953)
Dromedary	26	47	0.047 (0.010)	0.027 (0.130)	1.983 (0.922)	2.317 (0.941)

N, sample sizes; S, segregating sites; SSD, sum of squared deviations; Hapending’s r, Hapending’s Raggedness index.

1) Mismatch distribution did not converge after 2000 steps in Kazakhstan’s Bactrian camel population; therefore, haplotype H_2 from individual MH109985 was removed in this study.

## References

[1] Burger PA (2016). The history of Old World camelids in the light of molecular genetics. Trop Anim Health Prod.

[2] Saipolda T, Cardellino R, Rosati A, Mosconi C (2004). Mongolian camels.

[3] Burger PA, Ciani E, Faye B (2019). Old World camels in a modern world - a balancing act between conservation and genetic improvement. Anim Genet.

[4] Hare J (2008). Camelus ferus The IUCN red list of threatened species.

[5] Lei Y, Hare J, Guoying Y, Yun C, Knoll EM, Burger P (2012). The status of the wild camel in China. Camels in Asia and North Africa: interdisciplinary perspectives on their past and present significance.

[6] Yadamsuren A, Dulamtseren E, Reading RP, Knoll EM, Burger P (2012). The conservation status and management of wild camels in Mongolia. Camels in Asia and North Africa: interdisciplinary perspectives on their past and present significance.

[7] Al-Ali AK, Husayni HA, Power DM (1988). A comprehensive biochemical analysis of the blood of the camel (*Camelus dromedarius*). Comp Biochem Physiol B.

[8] Hamers-Casterman C, Atarhouch T, Muyldermans S (1993). Naturally occurring antibodies devoid of light chains. Nature.

[9] Wu H, Guang X, Al-Fageeh MB (2014). Camelid genomes reveal evolution and adaptation to desert environments. Nat Commun.

[10] Ming L, Wang Z, Yi L (2020). Chromosome-level assembly of wild Bactrian camel genome reveals organization of immune gene loci. Mol Ecol Resour.

[11] Ji R, Cui P, Ding F (2009). Monophyletic origin of domestic bactrian camel (*Camelus bactrianus*) and its evolutionary relationship with the extant wild camel (*Camelus bactrianus ferus*). Anim Genet.

[12] Mohandesan E, Fitak RR, Corander J (2017). Mitogenome sequencing in the genus *Camelus* reveals evidence for purifying selection and long-term divergence between wild and domestic Bactrian camels. Sci Rep.

[13] Cui P, Ji R, Ding F (2007). A complete mitochondrial genome sequence of the wild two-humped camel (*Camelus bactrianus ferus*): an evolutionary history of camelidae. BMC Genomics.

[14] Almathen F, Charruau P, Mohandesan E (2016). Ancient and modern DNA reveal dynamics of domestication and cross-continental dispersal of the dromedary. Proc Natl Acad Sci USA.

[15] Ming L, Yi L, Sa R, Wang ZX, Wang Z, Ji R (2017). Genetic diversity and phylogeographic structure of Bactrian camels shown by mitochondrial sequence variations. Anim Genet.

[16] Yi L, Ai Y, Ming L (2017). Molecular diversity and phylogenetic analysis of domestic and wild Bactrian camel populations based on the mitochondrial *ATP8* and *ATP6* genes. Livest Sci.

[17] Chuluunbat B, Charruau P, Silbermayr K, Khorloojav T, Burger PA (2014). Genetic diversity and population structure of Mongolian domestic Bactrian camels (*Camelus Bactrianus*). Anim Genet.

[18] Mate ML, Rocco FD, Zambelli A, Vidal-Rioja L (2004). Mitochondrial DNA structure and organization of the control region of South American camelids. Mol Ecol Notes.

[19] Silbermayr K, Orozco-terWengel P, Charruau P (2010). High mitochondrial differentiation levels between wild and domestic Bactrian camels: a basis for rapid detection of maternal hybridization. Anim Genet.

[20] Tamura K, Stecher G, Peterson D, Filipski A, Kumar S (2013). MEGA6: molecular evolutionary genetics analysis version 6.0. Mol Biol Evol.

[21] Librado P, Rozas J (2009). DnaSP v5: a software for comprehensive analysis of DNA polymorphism data. Bioinformatics.

[22] Bandelt HJ, Forster P, Röhl A (1999). Median-joining networks for inferring intraspecific phylogenies. Mol Biol Evol.

[23] Rogers AR, Harpending H (1992). Population growth makes waves in the distribution of pairwise genetic differences. Mol Biol Evol.

[24] Harpending HC (1994). Signature of ancient population growth in a low-resolution mitochondrial DNA mismatch distribution. Hum Biol.

[25] Fu YX (1997). Statistical tests of neutrality of mutations against population growth, hitchhiking and background selection. Genetics.

[26] Tajima F (1989). Statistical method for testing the neutral mutation hypothesis by DNA polymorphism. Genetics.

[27] Excoffier L, Lischer HE (2010). Arlequin suite ver 3.5: a new series of programs to perform population genetics analyses under Linux and windows. Mol Ecol Resour.

[28] Al-Jumaili AS, Boudali SF, Kebede A (2020). The maternal origin of indigenous domestic chicken from the Middle East, the north and the horn of Africa. BMC Genetics.

[29] Ramos-Onsins SE, Rozas J (2002). Statistical properties of new neutrality tests against population growth. Mol Biol Evol.

[30] Rogers AR, Harpending H (1992). Population growth makes waves in the distribution of pairwise genetic differences. Mol Biol Evol.

[31] Ming L, Yi L, Guo FC, Siriguleng S, Jirimutu J (2016). Molecular phylogeny of the Bactrian camel based on mitochondrial *cytochrome b* gene sequences. Genet Mol Res.

[32] Ming L, Yuan L, Yi L (2020). Whole-genome sequencing of 128 camels across Asia reveals origin and migration of domestic. Commun Biol.

