# Supplementary material for: Mitochondrial DNA variation and phylogeography of Old World camels
Source: Anim Biosci. 2020 Aug 24;34(4):525–32. doi: 10.5713/ajas.20.0319 (PMC7961272; doi:10.5713/ajas.20.0319)
Supplement: Supplementary file 1 [file ajas-20-0319-suppl.pdf]

Supplementary Materials

Table S1. Sequence information from GenBank.

No.	GenBank accession	Species	Origin
	**Domestic bactrian camel**		
	**China**		
1	MH109872	*Camelus bactrianus*	Qinghai, China
2	MH109873	*Camelus bactrianus*	Qinghai, China
3	MH109874	*Camelus bactrianus*	Qinghai, China
4	MH109875	*Camelus bactrianus*	Qinghai, China
5	MH109876	*Camelus bactrianus*	Qinghai, China
6	MH109877	*Camelus bactrianus*	Qinghai, China
7	MH109878	*Camelus bactrianus*	Qinghai, China
8	MH109879	*Camelus bactrianus*	Qinghai, China
9	MH109880	*Camelus bactrianus*	Qinghai, China
10	MH109881	*Camelus bactrianus*	Qinghai, China
11	MH109882	*Camelus bactrianus*	Alashan, Inner Mongolia
12	MH109883	*Camelus bactrianus*	Alashan, Inner Mongolia
13	MH109884	*Camelus bactrianus*	Alashan, Inner Mongolia
14	MH109885	*Camelus bactrianus*	Alashan, Inner Mongolia
15	MH109886	*Camelus bactrianus*	Alashan, Inner Mongolia
16	MH109887	*Camelus bactrianus*	Alashan, Inner Mongolia
17	MH109888	*Camelus bactrianus*	Alashan, Inner Mongolia
18	MH109889	*Camelus bactrianus*	Alashan, Inner Mongolia
19	MH109890	*Camelus bactrianus*	Alashan, Inner Mongolia
20	MH109891	*Camelus bactrianus*	Bayannaoer, Inner Mongolia
21	MH109892	*Camelus bactrianus*	Bayannaoer, Inner Mongolia
22	MH109893	*Camelus bactrianus*	Bayannaoer, Inner Mongolia
23	MH109894	*Camelus bactrianus*	Bayannaoer, Inner Mongolia
24	MH109895	*Camelus bactrianus*	Bayannaoer, Inner Mongolia
25	MH109896	*Camelus bactrianus*	Bayannaoer, Inner Mongolia
26	MH109897	*Camelus bactrianus*	Bayannaoer, Inner Mongolia
27	MH109898	*Camelus bactrianus*	Bayannaoer, Inner Mongolia
28	MH109899	*Camelus bactrianus*	Bayannaoer, Inner Mongolia
29	MH109900	*Camelus bactrianus*	Bayannaoer, Inner Mongolia

30	MH109901	*Camelus bactrianus*	Bayannaoer, Inner Mongolia
31	MH109902	*Camelus bactrianus*	Sunite, Inner Mongolia
32	MH109903	*Camelus bactrianus*	Sunite, Inner Mongolia
33	MH109904	*Camelus bactrianus*	Sunite, Inner Mongolia
34	MH109905	*Camelus bactrianus*	Sunite, Inner Mongolia
35	MH109906	*Camelus bactrianus*	Sunite, Inner Mongolia
36	MH109907	*Camelus bactrianus*	Sunite, Inner Mongolia
37	MH109908	*Camelus bactrianus*	Sunite, Inner Mongolia
38	MH109909	*Camelus bactrianus*	Sunite, Inner Mongolia
39	MH109910	*Camelus bactrianus*	Sunite, Inner Mongolia
40	MH109911	*Camelus bactrianus*	Sunite, Inner Mongolia
41	MH109931	*Camelus bactrianus*	Zhungeer, Xinjiang
42	MH109932	*Camelus bactrianus*	Zhungeer, Xinjiang
43	MH109933	*Camelus bactrianus*	Zhungeer, Xinjiang
44	MH109934	*Camelus bactrianus*	Zhungeer, Xinjiang
45	MH109935	*Camelus bactrianus*	Zhungeer, Xinjiang
46	MH109936	*Camelus bactrianus*	Mulei, Xinjiang
47	MH109937	*Camelus bactrianus*	Mulei, Xinjiang
48	MH109938	*Camelus bactrianus*	Mulei, Xinjiang
49	MH109939	*Camelus bactrianus*	Mulei, Xinjiang
50	MH109940	*Camelus bactrianus*	Mulei, Xinjiang
51	MH109941	*Camelus bactrianus*	Talimu, Xinjiang
52	MH109942	*Camelus bactrianus*	Talimu, Xinjiang
53	MH109943	*Camelus bactrianus*	Talimu, Xinjiang
54	MH109944	*Camelus bactrianus*	Talimu, Xinjiang
55	MH109945	*Camelus bactrianus*	Talimu, Xinjiang
56	NC_009628	*Camelus bactrianus*	China
57	EF212037	*Camelus bactrianus*	China
	**Mongolia**		
1	EF507798	*Camelus bactrianus*	Umnugobi, Mongolia
2	EF507799	*Camelus bactrianus*	Gobi-Altai, Mongolia
3	FJ792680	*Camelus bactrianus*	Mongolia
4	FJ792681	*Camelus bactrianus*	Mongolia
5	FJ792682	*Camelus bactrianus*	Mongolia

6	FJ792683	*Camelus bactrianus*	Mongolia
7	KF640722	*Camelus bactrianus*	Mongolia
8	KF640723	*Camelus bactrianus*	Mongolia
9	KF640724	*Camelus bactrianus*	Mongolia
10	KF640725	*Camelus bactrianus*	Mongolia
11	KF640726	*Camelus bactrianus*	Mongolia
12	KF640727	*Camelus bactrianus*	Mongolia
13	KF640729	*Camelus bactrianus*	Mongolia
14	KF640730	*Camelus bactrianus*	Mongolia
15	KF640731	*Camelus bactrianus*	Mongolia
16	MH109946	*Camelus bactrianus*	Hanbogd, Umnugobi
17	MH109947	*Camelus bactrianus*	Hanbogd, Umnugobi, Mongolia
18	MH109948	*Camelus bactrianus*	Hanbogd, Umnugobi
19	MH109949	*Camelus bactrianus*	Hanbogd, Umnugobi
20	MH109950	*Camelus bactrianus*	Hanbogd, Umnugobi
21	MH109951	*Camelus bactrianus*	Hanbogd, Umnugobi
22	MH109952	*Camelus bactrianus*	Hanbogd, Umnugobi
23	MH109953	*Camelus bactrianus*	Hanbogd, Umnugobi
24	MH109954	*Camelus bactrianus*	Mandal-ovoo, Umnugobi
25	MH109955	*Camelus bactrianus*	Mandal-ovoo, Umnugobi
26	MH109956	*Camelus bactrianus*	Mandal-ovoo, Umnugobi
27	MH109957	*Camelus bactrianus*	Mandal-ovoo, Umnugobi
28	MH109958	*Camelus bactrianus*	Mandal-ovoo, Umnugobi
29	MH109959	*Camelus bactrianus*	Mandal-ovoo, Umnugobi
30	MH109960	*Camelus bactrianus*	Mandal-ovoo, Umnugobi
31	MH109961	*Camelus bactrianus*	Mandal-ovoo, Umnugobi
32	MH109962	*Camelus bactrianus*	Mandal-ovoo, Umnugobi
33	MH109963	*Camelus bactrianus*	Mandal-ovoo, Umnugobi
34	MH109964	*Camelus bactrianus*	Tugrug, Gobi Altai
35	MH109965	*Camelus bactrianus*	Tugrug, Gobi Altai
36	MH109966	*Camelus bactrianus*	Tugrug, Gobi Altai
37	MH109967	*Camelus bactrianus*	Tugrug, Gobi Altai
38	MH109968	*Camelus bactrianus*	Tugrug, Gobi Altai
39	MH109969	*Camelus bactrianus*	Tugrug, Gobi Altai
40	MH109970	*Camelus bactrianus*	Tugrug, Gobi Altai

41	MH109971	*Camelus bactrianus*	Tugrug, Gobi Altai
42	MH109972	*Camelus bactrianus*	Tugrug, Gobi Altai
43	MH109973	*Camelus bactrianus*	Tugrug, Gobi Altai
	**Kazakhstan**		
1	MH109984	*Camelus bactrianus*	Kazakhstan
2	MH109985	*Camelus bactrianus*	Kazakhstan
3	MH109986	*Camelus bactrianus*	Kazakhstan
4	MH109987	*Camelus bactrianus*	Kazakhstan
5	MH109988	*Camelus bactrianus*	Kazakhstan
6	MH109989	*Camelus bactrianus*	Kazakhstan
	**Russia**		
1	MH109974	*Camelus bactrianus*	Astrakhan, Russia
2	MH109975	*Camelus bactrianus*	Astrakhan, Russia
3	MH109976	*Camelus bactrianus*	Astrakhan, Russia
4	MH109977	*Camelus bactrianus*	Astrakhan, Russia
5	MH109978	*Camelus bactrianus*	Astrakhan, Russia
6	MH109979	*Camelus bactrianus*	Astrakhan, Russia
7	MH109980	*Camelus bactrianus*	Astrakhan, Russia
8	MH109981	*Camelus bactrianus*	Astrakhan, Russia
9	MH109982	*Camelus bactrianus*	Astrakhan, Russia
10	MH109983	*Camelus bactrianus*	Astrakhan, Russia
	**IRAN**		
1	KX554925	*Camelus bactrianus*	Iran
2	KX554927	*Camelus bactrianus*	Iran
3	KX554928	*Camelus bactrianus*	Iran
4	KX554929	*Camelus bactrianus*	Iran
5	KX554930	*Camelus bactrianus*	Iran
6	MH109990	*Camelus bactrianus*	Iran
7	MH109991	*Camelus bactrianus*	Iran
8	MH109992	*Camelus bactrianus*	Ardabili, Iran
9	MH109993	*Camelus bactrianus*	Ardabili, Iran
10	MH109994	*Camelus bactrianus*	Ardabili, Iran

11	MH109995	*Camelus bactrianus*	Ardabili, Iran
12	MH109996	*Camelus bactrianus*	Ardabili, Iran
13	MH109997	*Camelus bactrianus*	Ardabili, Iran
	**Wild**		
1	NC_009629	*Camelus ferus*	Gobi-Altai, Mongolia
2	EF212038	*Camelus ferus*	Gobi-Altai, Mongolia
3	EF507801	*Camelus ferus*	Gobi-Altai, Mongolia
4	EF507800	*Camelus ferus*	Gobi-Altai, Mongolia
5	FJ792684	*Camelus ferus*	Gobi-Altai Mongolia
6	FJ792685	*Camelus ferus*	Gobi-Altai, Mongolia
7	MH109912	*Camelus ferus*	Gobi-Altai, Mongolia
8	MH109913	*Camelus ferus*	Gobi-Altai, Mongolia
9	MH109914	*Camelus ferus*	Gobi-Altai, Mongolia
10	MH109915	*Camelus ferus*	Gobi-Altai, Mongolia
11	MH109916	*Camelus ferus*	Gobi-Altai, Mongolia
12	MH109917	*Camelus ferus*	Gobi-Altai Mongolia
13	MH109918	*Camelus ferus*	Gobi-Altai, Mongolia
14	MH109919	*Camelus ferus*	Gobi-Altai, Mongolia
15	MH109920	*Camelus ferus*	Gobi-Altai, Mongolia
16	MH109921	*Camelus ferus*	Gobi-Altai, Mongolia
17	MH109922	*Camelus ferus*	Gobi-Altai, Mongolia
18	MH109923	*Camelus ferus*	Gobi-Altai, Mongolia
19	MH109924	*Camelus ferus*	Gobi-Altai Mongolia
20	MH109925	*Camelus ferus*	Gobi-Altai, Mongolia
21	MH109926	*Camelus ferus*	Gobi-Altai, Mongolia
22	NM109927	*Camelus ferus*	Gobi-Altai, Mongolia
23	MH109928	*Camelus ferus*	Gobi-Altai, Mongolia
24	NM109929	*Camelus ferus*	Gobi-Altai, Mongolia
25	MH109930	*Camelus ferus*	Gobi-Altai, Mongolia
26	Hybrid*	*Camelus ferus*	Gobi-Altai Mongolia
27	Hybrid*	*Camelus ferus*	Gobi-Altai, Mongolia
	**Dromedary**		
1	MH109998	*Camelus dromedarius*	Ardabili, Iran
2	MH109999	*Camelus dromedarius*	Ardabili, Iran
3	MH110000	*Camelus dromedarius*	Ardabili, Iran

4	MH110003	*Camelus dromedarius*	Ardabili, Iran
5	MH110004	*Camelus dromedarius*	Ardabili, Iran
6	MH110005	*Camelus dromedarius*	Ardabili, Iran
7	MH110001	*Camelus dromedarius*	Ardabili, Iran
8	MH110002	*Camelus dromedarius*	Ardabili, Iran
9	MH110006	*Camelus dromedarius*	Ardabili, Iran
10	MH110007	*Camelus dromedarius*	Ardabili, Iran
11	KX554931	*Camelus dromedarius*	Iran
12	KX554932	*Camelus dromedarius*	Iran
13	KX554933	*Camelus dromedarius*	Iran
14	KX554934	*Camelus dromedarius*	Iran
15	KU605072	*Camelus dromedarius*	Qatar, Jordan border
16	KU605073	*Camelus dromedarius*	Saudi Arabia
17	KU605074	*Camelus dromedarius*	Saudi Arabia
18	KU605075	*Camelus dromedarius*	Saudi Arabia
19	KU605076	*Camelus dromedarius*	Saudi Arabia
20	KU605077	*Camelus dromedarius*	United Arab Emirates, Dubai
21	KU605078	*Camelus dromedarius*	Kenya
22	KU605079	*Camelus dromedarius*	Sudan
23	KU605080	*Camelus dromedarius*	Pakistan
24	JN632608	*Camelus dromedarius*	Morocco
25	EU159113	*Camelus dromedarius*	United Arab Emirates, Dubai
26	NC_009849	*Camelus dromedarius*	United Arab Emirates, Dubai

"*" : Hybrids of domestic and wild bactrian camels from Liang et al[15].

Table S2. Haplotypes that were shared between domestic Bactrian camel populations from five countries.

Shared haplotype	No. of sequence	Country	Number
		Kazakhstan	1
H_1	7	China	2
		Mongolia	2
		Kazakhstan	4
H_3	21	China	11
		Mongolia	6
		Iran	7
H_4	45	China	26
		Mongolia	12
		Iran	4
H_6	6	China	1
		Mongolia	1
H_15	5	China	4
		Mongolia	1
H_16	16	China	8
		Mongolia	8

Figure S1. Polymorphic sites and distribution of haplotypes from domestic (H_1 - H_30) and wild Bactrian camels (W_1 and W_2). The vertical numbers indicate the position of the polymorphism compared to the mtDNA sequence of reference (GenBank accession no. NC_009628). The right column under "N" shows the number of times a haplotype was observed. Dots (.) indicate that the same nucleotide compared to the reference sequences was found, and the letters show the polymorphism. H represent haplotypes from domestic bactrian camels, and W represent haplotypes from wild Bactrian camels.

.

```
              11111122222222333333333333445555555555666777778
       356789924446902234579133455667789380234477999046445990
       995747880234421862427712512787904874407839067370034560  N
H_1    AAGGATGGTTGTCGAATTACCCGGAACGACCATATACAATCGCCAGACTGACTA  7
H_2    ..A....AC.A..AGGC.G.TT.A........C..G..C....T..T.CAGT..  1
H_3    .................................GC...................  21
H_4    ..................................C...................  45
H_5    ..................................C......T............  1
H_6    GT.......C............................................  6
H_7    .....C................................C....T......GT..  1
H_8    .GA.GCA....C.AGGC.G.TTAA.T.C..TGC....GC....T..T.CAGT..  1
H_9    .GA.GCA....C......................C..GC...............  5
H_10   .GA.GCA....C.....................GC..GC...............  1
H_11   .GA.GCA....C.................T....C..GC...............  1
H_12   .GA.GCA....C.........................GC...............  1
H_13   .GA.GCA....C...............A......C..GC...............  1
H_14   .T....................................................  1
H_15   ...............................G..C...................  5
H_16   GT....................................................  16
H_17   .................................G....................  1
H_18   ..................................C....C..............  2
H_19   GT..........................................G.........  1
H_20   GT.................T..................................  1
H_21   ............................G.....C...................  1
H_22   ..................................C.....T.A..T........  1
H_23   .................................GC....C..............  1
H_24   GT.....................................C..............  1
H_25   .................C................C...................  1
H_26   .......................................C..............  1
H_27   .................................G.....C..............  1
H_28   GT................................C...................  1
H_29   ..................................C.................CT  1
H_30   ...A..............................C...................  1
W_1    .GA.GCA....CT...........G.T..........GC........T..G...  9
W_1    .GA.GCA.....T...........G.T.........TGC........T..G...  18
```

Figure S2. Polymorphic sites and distribution of haplotypes from dromedaries. The vertical numbers indicate the position of the polymorphism compared to the mtDNA sequence of reference (GenBank accession no. NC_009849). The right column under “N” shows the number of times a haplotype was observed. Dots (.) indicate that the same nucleotide compared to the reference sequences was found, and the letters show the polymorphism.

```
                       11111122222223333333333333355778
        1224555677889912446902223591233455677788909440
       21094369247497818034421896477012512701901447036  N
D_1    TGATGTGAACAATTGTACATCAGGGCGTTGAAATCCGTTGGCGACAG  7
D_2    ........................................A......  1
D_3    .............................................G.  1
D_4    ....A...................A.....G.....A.....A...C  1
D_5    .............................AT............G.G.  1
D_6    ..........................................A....  1
D_7    ...............G...............................  2
D_8    ...............G............................T.C  2
D_9    CAGCACAGGTGGCCA.GTG..GAA.TACC.GG.A.GAACAA......  4
D_10   CAGCACAGGTGGCCA.GTGC.GAA.TACC.GGGATGAACAA......  1
D_11   CAGCACAGGTGGCCA.GTG..GAA.TACC.GGGATGAACAA......  2
D_12   CAGCACA.GTGGC...GTG..GAA.TACC.GG.A.GAACAA......  1
D_13   CAGCACAGGTGGCCA.GTG..GAA.TACC.GG.A.GAACAATA....  1
D_14   CAGCACAGGTGGCCA.GTGCTGAA.TACC.GG.A.GAACAA......  1
```

Figure S3. Neighbor-joining tree for the 46 haplotypes from domestic and wild Bactrian camel populations, and dromedaries. Bootstrap values >50 were retained and are shown on the node. H represents haplotypes from domestic Bactrian camels, W represent haplotypes from wild Bactrian camels, and D represent haplotypes from dromedaries.

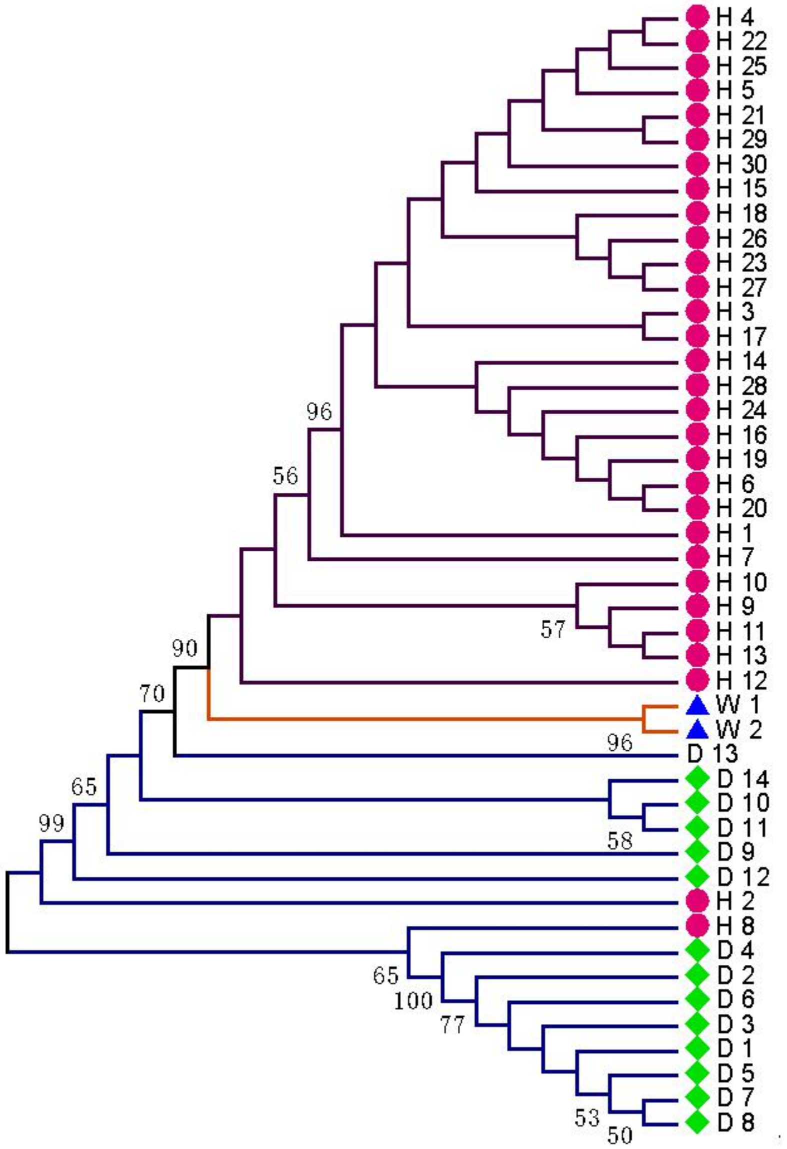

Figure S4. Median-joining network for all haplotypes in this study from domestic and wild Bactrian camels, and dromedaries. The area of the circle is proportional to haplotype frequency. All camel haplotypes were divided into three distinct lineages, and for haplotypes H_2 (MH109985) and H_8 (MH109974) from the Bactrian camels, rather than clustering with the Bactrian camel groups, they were closer to the Iranian dromedary.

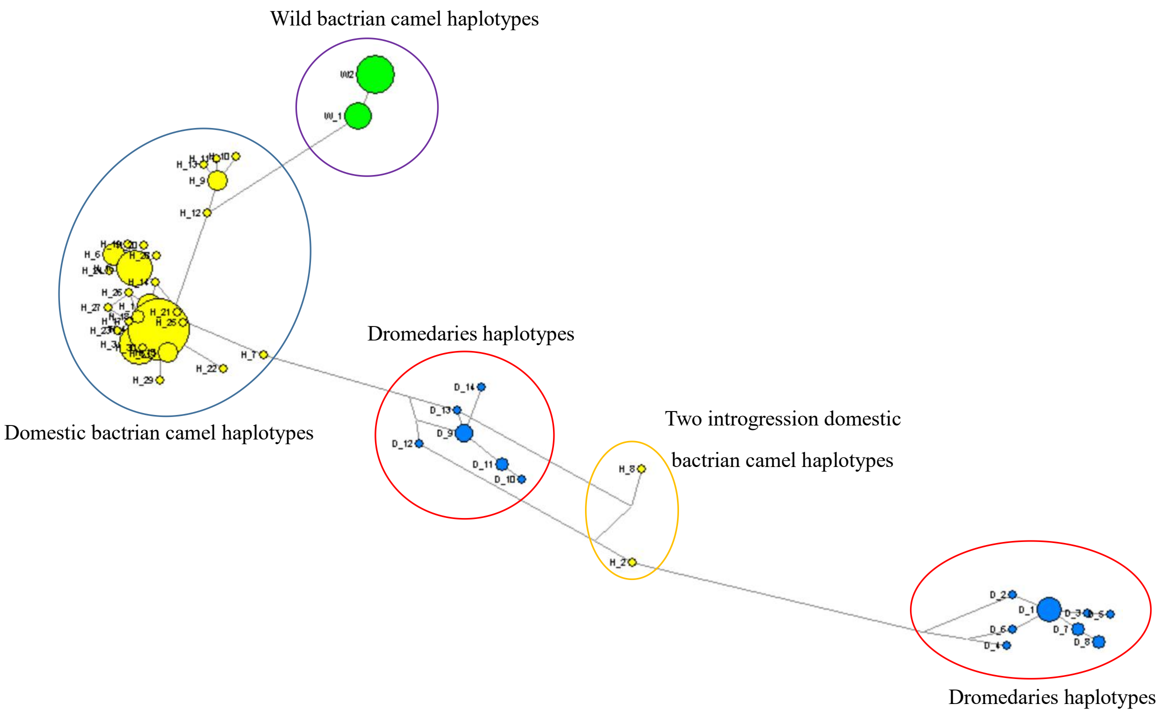

Figure S5. Mismatch distribution patterns for China, Mongolia, Russia, Kazakhstan, Iran, and all domestic bactrian camel. The abscissa is frequency and the ordinate is pairwise differences. The red lines indicate Freq.Obs., and the green lines indicate Freq.Exp. The mismatch distribution graphs revealed uni-, bi- and multi-modal patterns, which suggest that there has been a demographic expansion, bottleneck or purifying selection in different camel populations.

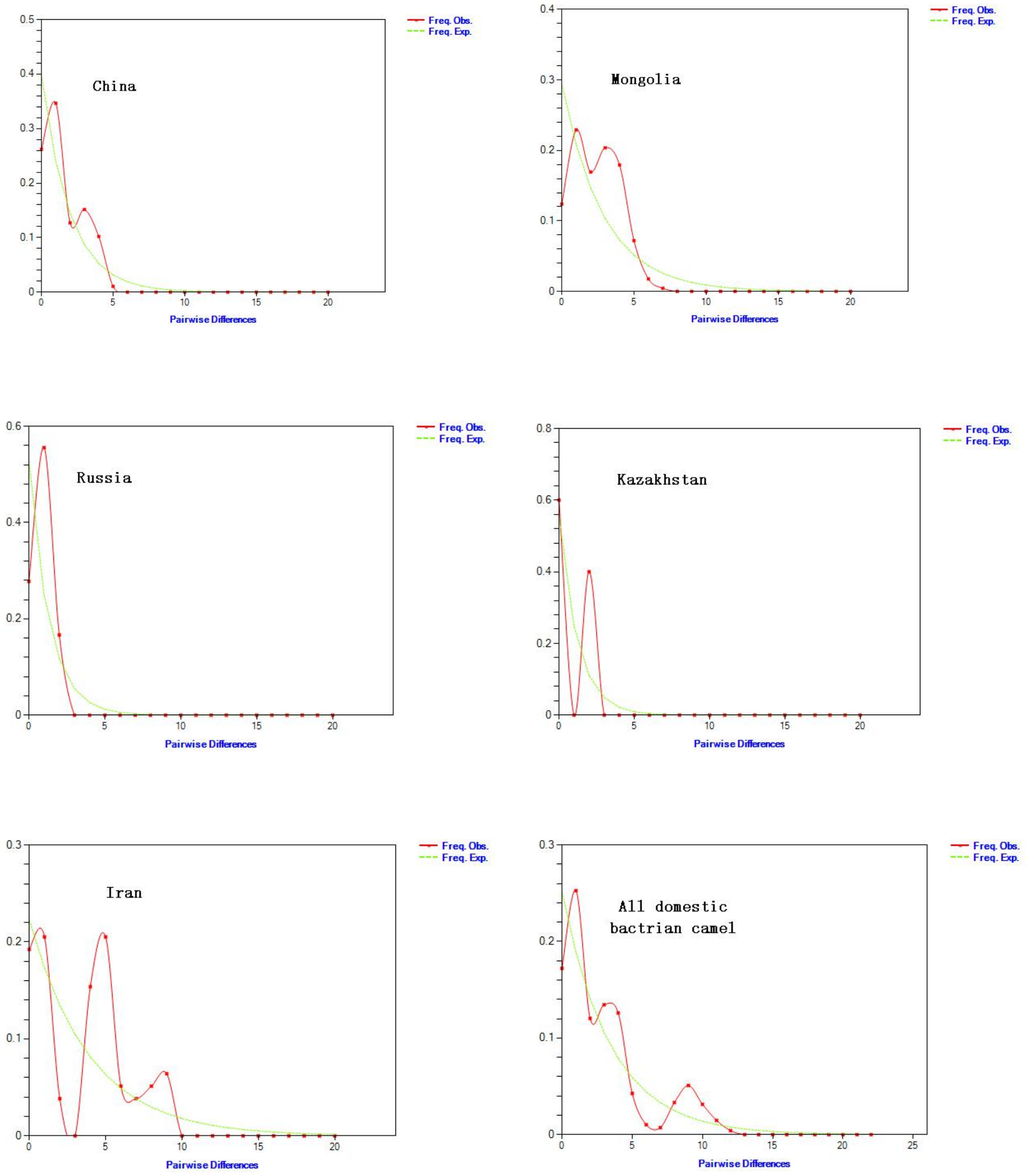

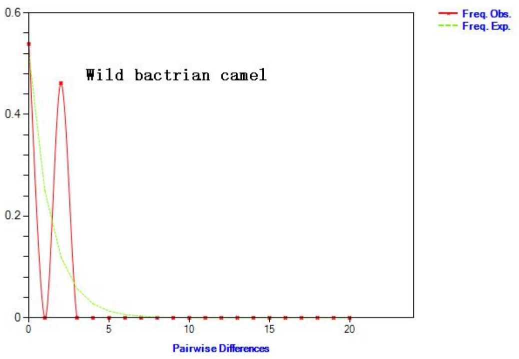
Wild bactrian camel
Freq. Obs.
Freq. Exp.
Pairwise Differences
0.6
0.4
0.2
0
0
5
10
15
20

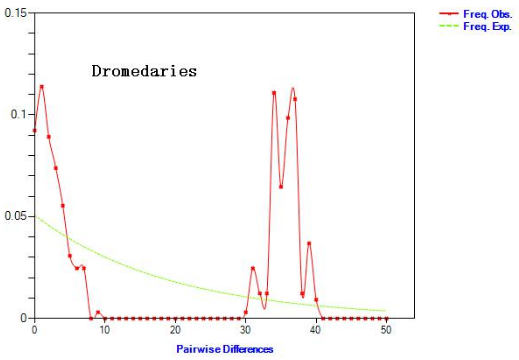
Dromedaries
Freq. Obs.
Freq. Exp.
Pairwise Differences
0.15
0.1
0.05
0
0
10
20
30
40
50
